# Regulation of fleshy fruit ripening: from transcription factors to epigenetic modifications

**DOI:** 10.1093/hr/uhac013

**Published:** 2022-02-11

**Authors:** Xiuming Li, Xuemei Wang, Yi Zhang, Aihong Zhang, Chun-Xiang You

**Affiliations:** 1 National Key Laboratory of Crop Biology, Shandong Collaborative Innovation Center of Fruit & Vegetable Quality and Efficient Production, College of Horticulture Science and Engineering, Shandong Agricultural University, Tai-An, Shandong 271018, China; 2 Shandong Provincial Key Laboratory of Plant Stress, College of Life Sciences, Shandong Normal University, Jinan 250014, China; 3 State Key Laboratory of Crop Biology, College of Life Sciences, Shandong Agricultural University, Tai-An, 271018, China

## Abstract

Fleshy fruits undergo a complex ripening process, developing organoleptic fruit traits that attract herbivores and maximize seed dispersal. Ripening is the terminal stage of fruit development and involves a series of physiological and biochemical changes. In fleshy fruits, ripening always involves a drastic color change triggered by the accumulation of pigments and degradation of chlorophyll, softening caused by cell wall remodeling, and flavor formation as acids and sugars accumulate alongside volatile compounds. The mechanisms underlying fruit ripening rely on the orchestration of ripening-related transcription factors, plant hormones, and epigenetic modifications. In this review, we discuss current knowledge of the transcription factors that regulate ripening in conjunction with ethylene and environmental signals (light and temperature) in the model plant tomato (*Solanum lycopersicum*) and other fleshy fruits. We emphasize the critical roles of epigenetic regulation, including DNA methylation and histone modification as well as RNA m^**6**^A modification, which has been studied intensively. This detailed review was compiled to provide a comprehensive description of the regulatory mechanisms of fruit ripening and guide new strategies for its effective manipulation.

## Introduction

Fruits of angiosperm plants represent organs specialized for the protection of developing seeds and, upon ripening, for maximizing the dispersal of mature seeds. Different plant species have evolved a variety of distinct strategies for seed dispersal to ensure the survival of the next generation. To accommodate their changing functions, fruits undergo profound physiological changes during their development that can typically be classified into three stages: fruit set, growth, and ripening.

Ripening processes of fruits have a remarkable impact on their quality and consumer acceptance. Fruit ripening, the terminal stage of fruit development, is a complex process involving a series of physiological and biochemical changes. In fleshy fruits, ripening always involves drastic changes in color as pigments accumulate and chlorophylls degrade, fruit softening as cell walls are remodeled, and flavor development as acids, sugars, and volatile compounds accumulate. These changes vary among species to create fleshy fruits with an impressive variety of flavors and forms [1–3]. Fleshy fruits can be further divided into two groups: climacteric and non-climacteric. Climacteric fruits, such as tomato, apple (*Malus domestica*), and banana (*Musa acuminata*), display a burst of respiration and a rapid increase in ethylene accumulation at the initiation of fruit ripening, whereas ripening of non-climacteric fruits is less reliant on elevated ethylene levels. Furthermore, other hormones control specific features of the fruit ripening process. For example, auxin facilitates the shift from growth to ripening, and abscisic acid (ABA) serves as a major fruit ripening and senescence regulator [4].

The fruit ripening program is coordinated by the combined effects of plant hormones, transcription factors (TFs), and epigenetic modifications, all of which can influence fruit quality [5, 6]. Given the crucial role of fleshy fruit in the human diet, there has been growing interest over the past two decades in exploring the mechanisms underlying fruit ripening using physiological and genetic approaches. The molecular basis of hormonal activity and crosstalk during fruit ripening has been discussed in previous reviews [3, 4, 7, 8]. Here, we focus on recent advances in elucidating the crucial roles of TF crosstalk with hormonal activity, especially ethylene, and epigenetic modifications in regulating fruit ripening in the key fleshy fruit model tomato and additional species.

**Figure 1:**
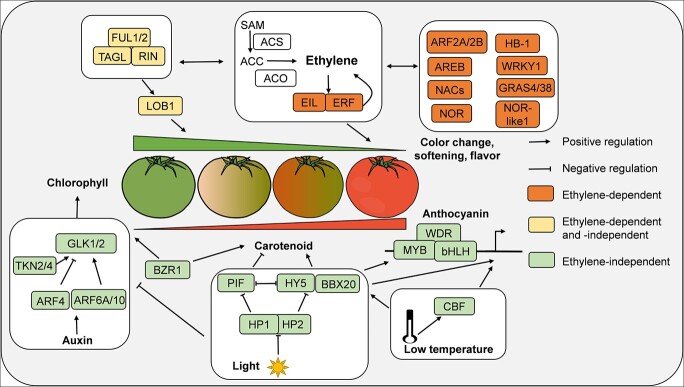
Schematic representation of ripening-related TFs interacting with environmental and hormonal signals to regulate tomato fruit ripening. Rounded rectangles represent TFs and signaling components. Ethylene-dependent TFs are shown in bright orange, ethylene-independent TFs are shown in light green, while ethylene-dependent and -independent TFs are shown in light yellow. The ripening master TFs RIN, TAGL, and FUL1/2 act together with ethylene to regulate almost every aspect of ripening. Meanwhile, RIN regulates fruit softening by directly targeting LOB1. TFs such as HB-1, NAC, NOR, NOR-like1, GRAS4/38, AREB, ARF2A/2B, and WRKY1 regulate fruit ripening by altering ethylene biosynthesis and responses. Carotenoid and chlorophyll contents during tomato ripening are indicated by green and red triangles, respectively. Chlorophyll accumulation and degradation is regulated by several TFs, including GLK1/2, TKN2/4, and ARFs, and this process is modulated by the interplay of auxin and light. BZR1, a brassinosteroid response TF, regulates chlorophyll accumulation via promoting *GLK2* expression. Light promotes fruit colorization through HY5 and BBX20 proteins, and low temperatures regulate anthocyanin accumulation in a light-dependent signaling pathway and also in a way connecting with cold-induced CBF signaling.

## Transcription factors in fruit ripening

### Ethylene biosynthesis, perception, and signaling transduction

Ethylene induces a well-organized signaling pathway that coordinates plant growth and development and fruit ripening [9]. In climacteric fruits, ethylene plays a predominant role at both the immature fruit stage and the ripening stage [6, 7]. Immature fruits maintain a basal content of ethylene; exogenous ethylene does not stimulate fruit ripening and can even delay ripening. This stage is defined as System 1. At the onset of fruit ripening, a large amount of ethylene is abruptly biosynthesized, facilitating the initiation of fruit ripening; this is characterized as System 2 [5–7]. The molecular signals that cause the change from System 1 to System 2 remain unclear. Genes involved in ethylene biosynthesis, reception, and downstream signal transduction have been identified in vascular plants [9]. Ethylene biosynthesis results from the action of two key enzymes: ACC synthase (ACS), which catalyzes the conversion of *S*-adenosyl-l-Met (SAM) to 1-amino-cyclopropane-1-carboxylic acid (ACC), and ACC oxidase (ACO), which catalyzes the conversion of ACC to ethylene [5, 7]. Changes in the expression of different members of the *ACS* and *ACO* gene families determine fruit developmental stages [7, 10]. Ethylene is perceived by ethylene receptor (ETR) proteins and activates a downstream transcriptional regulatory cascade including two families of ethylene-responsive TFs, ETHYLENE-INSENSITIVE3 (EIN3)-Like (EIL) and ETHYLENE RESPONSE FACTOR (ERF) [7]. ERF TFs transcriptionally activate the ethylene-inducible genes by directly binding to the GCC box in the promoters [11]. In tomato, mutation of ethylene biosynthesis genes, receptor genes, and response genes results in compromised ripening phenotypes [7].

It is widely accepted that ethylene, together with other endogenous plant hormones, works with TFs to coordinately regulate fruit ripening [3–7]. Several compelling lines of evidence suggest that interactions between ethylene and other plant hormones during the regulation of fruit ripening commonly converge at ripening-related TFs [12–14]. Here, we characterize the molecular mechanisms of TFs that regulate fruit ripening in ethylene-dependent and/or -independent manners (Fig. 1).

### Transcriptional regulation of fruit ripening mainly in an ethylene-dependent manner

The spontaneous ripening-related monogenic tomato mutants *rin* (*ripening-inhibitor*), *nor* (*non-ripening*), and *cnr* (*colorless non-ripening*) exhibit complete non-ripening phenotypes; thus, the genes affected in the mutants were previously considered to be master regulators of fruit ripening. *RIN* encodes a MADS box TF, while *NOR* and *CNR* encode NAC and SQUAMOSA promoter binding protein-like (SPB) TFs, respectively [15–17]. The roles of these three genes in regulating fruit ripening were recently reconsidered after the generation of null mutant alleles using CRISPR/Cas9 technology. The *rin-ko*, *nor-ko*, and *cnr-ko* mutants exhibit partial non-ripening phenotypes compared with their respective original mutants, suggesting that the traditional *rin*, *nor*, and *cnr* mutations resulted from dominant negative mutations or gain-of-function mutations [18–21]. Numerous homologous members of the MADS-RIN and NAC families involved in fruit ripening have been identified in tomato and other fleshy fruit species, providing evidence that traditional *rin* and *nor* mutants display dominant negative activities that inhibit the transcriptional activity of their paralogs or directly repress the expression of downstream target genes. Furthermore, these observations suggest that RIN, NOR, and their paralogs are partially redundant [6, 8, 22].

Fruits of the *rin* mutant are green when mature; these fruits are insensitive to ethylene and do not turn red. MADS-RIN protein binds to the promoters of most ripening-related genes, including the ethylene biosynthesis genes *ACS2*, *ACS4*, and *ACO1*, ethylene receptor genes *ETR3* and *ETR4*, *ERF* genes, and other key genes involved in fruit ripening (e.g. carotenoid biosynthesis, cell wall softening, sugar metabolism, and aroma biosynthesis genes) [23–25]. Furthermore, multiple studies have demonstrated that the MADS box family TFs TOMATO AGAMOUS 1 (TAG1), TOMATO AGAMOUS-LIKE 1 (TAGL1) [26, 27], and FRUITFULL homologs (FUL1 and FUL2) [28] are involved in controlling fruit ripening. TAGL1 acts as a positive regulator that controls fruit ripening by regulating ethylene biosynthesis. Knockdown of *TAGL1* reduces expression of *ACS2* [26, 27]. FUL1 and FUL2 were originally thought to regulate fruit ripening in an ethylene-independent manner since ethylene biosynthesis and perception appeared normal in *FUL1*/*2* RNAi fruits [28]. However, advanced studies revealed that FUL1/FUL2, TAGL1, and RIN interact with each other to form a tetrameric DNA-binding complex that binds to the promoters of ethylene biosynthesis genes, thereby regulating tomato fruit ripening [29]. MADS-RIN homologs in other climacteric fruit species, such as apple MdMADS8/9 [30] and banana MaMADS1/2 [31], also regulate fruit ripening through ethylene biosynthesis. In summary, MADS box TFs appear to be upstream regulators that control fruit ripening by regulating ethylene biosynthesis and response. The expression of MADS box ripening genes (*RIN*, *FUL1*, *FUL2*, and *TAGL1*) is induced by ethylene, suggesting that MADS box TFs and ethylene comprise a regulatory circuit controlling fruit ripening [22, 23].

*NOR* encodes an NAC (NAM/ATAF1/CUC2) TF with a 2-bp deletion at its C-terminal, resulting in a frameshift mutation that produces a truncated protein containing only the NAC domain; *NOR* retains the ability to bind to the promoters of downstream genes, but fails to activate their expression [19]. In tomato, the functions of *SlNAC1*, *SlNAC4*, *SNAC4*/*SlNAC48*, *SNAC9*/*SlNAC19*, and *NOR-Like* genes in regulating fruit ripening have been studied intensively [33–36]. SlNAC4, SNAC4, SNAC9, and NOR-Like positively regulate fruit ripening by upregulating ethylene- and ripening-associated genes [33, 35, 36]. SlNAC1 negatively controls fruit pigmentation and softening during ripening and has been shown to bind to the promoters of ethylene biosynthesis genes, inactivating their expression [34]. Several kiwifruit (*Actinidia* spp.) NAC TFs control ethylene-associated monoterpene production (AaNAC2/3/4) and ethylene biosynthesis (AaNAC6/7, AaNAC2/72) during fruit ripening [37–39]. MaNAC1 and MaNAC2 positively regulate banana fruit ripening by removing the inhibition by MaERF11, a negative regulator that recruits histone deacetylase MaHDA1 to inactivate *MaACS1* and *MaACO1* [40, 41]. These findings demonstrate that regulation of genes involved in ethylene biosynthesis and other aspects of ripening by NAC TFs is conserved in different fruits.

Fruits of the tomato *cnr* mutant have yellow skin, fail to soften, and exhibit reduced ethylene production. Positional cloning showed that the *CNR* promoter is hypermethylated, resulting in decreased expression of *CNR* and *ACO* in the *cnr* mutant [16]. This epimutant revealed the potential relationship between epigenetic regulation and fruit ripening. Many TFs identified in the last two decades, such as HD-zip homeobox protein (LeHB-1), SlWRKY1, and SlGRAS4, regulate fruit ripening through a similar mechanism by directly binding to the promoters of ethylene biosynthesis genes and upregulating their transcripts [42–44]. SlARF2A and SlARF2B from the auxin response factor family act redundantly to regulate fruit ripening. Downregulation of both genes severely inhibits fruit ripening, with strong downregulation of the expression of ethylene biosynthesis genes, such as *ACO1*, *ACS2*, *ACS3*, and *ACS4*, and a dramatic reduction in ethylene production [45]. SlAREB1, a TF that positively regulates fruit ripening, is an ABA-response element binding factor (AREB) in the ABA signaling pathway important for regulating the accumulation of sugars and organic acids in tomato [46]. Ethylene biosynthesis genes *SlACS2*, *SlACO1*, and *SlACO3* as well as genes associated with primary carbohydrate and amino acid metabolism are upregulated in plants overexpressing *SlAREB1* [46]. Mou *et al*. [47] also found that SlAREB1 mediates ABA-promoted ethylene biosynthesis through transcriptionally activating *NOR*.

### Transcriptional regulation of fruit ripening mainly in an ethylene-independent manner

The interplay of hormones and light signals is important for chlorophyll degradation and colorization during the ripening transition stage [48, 49]. Mutation of the *uniform ripening* (*u*) and *uniform gray-green* (*ug*) loci results in a light green fruit phenotype caused by reduced chlorophyll levels in the pericarp and impaired chloroplast development, indicating that U and UG are required for chlorophyll accumulation and chloroplast development in mature green fruit. The *U* locus encodes the Golden 2-like TF, SlGLK2, while the *UG* locus encodes the Class I Knotted1-like Homeobox TF, TNK4. TKN4 and its homolog TKN2 activate expression of *SlGLK2* to promote the development of chloroplasts in tomato fruit [50, 51]. The overexpression of *SlGLK2* produces similar ethylene emission to that in control fruits and has no influence on the expression of *RIN*, *CNR*, and *TAGL1*, indicating that SlGLK2 specifically regulates mature green fruit plastid activity and plastid numbers, including sugar and later carotenoid accumulation [52]. SlARF4 negatively regulates chlorophyll accumulation and chloroplast development in immature fruit specifically, probably by binding to and inactivating the promoter of *SlGLK1* [53]. Expression of *SlARF4* is induced by auxin and light but is not affected by ethylene, indicating that SlARF4-promoted chlorophyll degradation during ripening transition is triggered by auxin and light and independent of ethylene [53, 54]. By contrast, SlARF6A and SlARF10 positively regulate chlorophyll and sugar accumulation in tomato fruits by directly binding to the promoter of *SlGLK1* [55, 56]. Overexpression of *SlARF6A* reduces ethylene production, indicating that a low level of ethylene is important for chlorophyll accumulation. Additionally, jasmonic acid and brassinosteroid also control pigment accumulation in tomato fruits. The exogenous application of methyl jasmonate (MeJA) induces carotenoid accumulation in the fruits of ethylene receptor mutant *etr3* (also named as *Never ripe*, *Nr*) with an unknown mechanism [57]. Similar phenotypes were obtained when treated with 2,4-epibrassinolide (EBR) in *etr3* fruits [58]. Sequential experiments demonstrate that the brassinosteroid response TF Brassinazole resistant 1 (BZR1) positively regulates chlorophyll accumulation in mature green fruit and carotenoid accumulation in ripe red fruit by the upregulation of *SlGLK2* and the carotenoid biosynthesis gene *PHYTOENE SYNTHASE 1* (*SlPSY1*) [58]. These observations suggest that jasmonic acid and brassinosteroid might regulate carotenoid biosynthesis in an ethylene-independent way in tomato fruits.

Phytochrome interacting factor (PIF) and bZIP TF LONG HYPOCOTYL 5 (HY5) make up the regulatory module downstream of photoreceptors coordinating light-associated fruit ripening responses [59]. In tomato, SlPIFs maintain the presence of chlorophyll in mature green fruit pericarp and inhibit the accumulation of carotenoids [60, 61]. When fruit ripening is initiated, light triggers degradation of SlPIFs by photoreceptor phytochrome, causing degradation of chlorophyll [60, 61]. SlHY5 is a potent PIF antagonist that promotes tomato fruit carotenoid and anthocyanin accumulation by directly binding to the promoters of pigment biosynthesis genes and activating their expression in response to light [62, 63]. Moreover, SlHY5 also regulates transcription of ethylene biosynthetic and response genes, such as *ACS2* and *ERF.E1* [63]. These results suggest that SlHY5 might function partially dependent on ethylene to regulate fruit colorization [63]. Light regulation of anthocyanin accumulation in other fruit crops, such as apple [64], grape (*Vitis vinifera*) [65], pear (*Pyrus pyrifolia*) [66], and peach (*Prunus persica*) [67], has been studied intensively. These fruits employ similar regulatory mechanisms to tomato, with HY5 proteins positively regulating transcription of downstream anthocyanin biosynthetic genes as well as the MYB-bHLH-WD40 Repeat (MBW) TF complex (a conserved regulatory module that regulates expression of anthocyanin biosynthesis structural genes). Tomato high pigment proteins HP1 (UV-DAMAGED DNA BINDING PROTEIN1, DDB1) and HP2 (DEETIOLATED1, DET1) act as suppressors of light signaling components that interact with SlHY5 to degrade its protein levels in the dark through CULLIN4 (CUL4)-RING ubiquitin E3 ligases (CRL4s) [49]. The fruits of light-hyperresponsive *hp1* and *hp2* mutants possess increased numbers and sizes of chloroplasts in mature green fruit and enhanced flavonoid, lycopene, and β-carotene accumulation in ripe red fruit [68, 69]. As mentioned above, SlGLK2 and TNK4 positively regulate chlorophyll accumulation in mature green fruit. A high level of chlorophyll in mature green fruit of the *hp1* mutant is partially caused by high expression levels of these two genes [50, 51]. Tang *et al*. [70] further proved that SlGLK2 protein stability is promoted in the *hp1* mutant and SlGLK2 is subject to HP1-based degradation via CRL4s. As in *SlARF6A* overexpression fruits, the ethylene content in *hp1* and *hp2* mutants is reduced in mature green fruit, indicating that light and auxin rather than ethylene might play predominant roles in chlorophyll accumulation in mature green fruit [56, 68, 69]. The high levels of carotenoids and anthocyanins in *hp1* and *hp2* ripe red fruit are probably due to the overaction of SlHY5 protein and other pigment biosynthesis regulators such as B-box zinc finger TF SlBBX20 [49, 71]. It must be pointed out that the impeded fruit softening of the *hp1* mutant may be caused by low content of ethylene and downregulation of cell wall-related genes [69]. The interplay of light and auxin is important for chlorophyll accumulation and breakdown [53, 54, 56, 68]. However, the non-specific and complex roles of these signals make it unclear which factors trigger ripening transition, in which tissues hormones modulate the ripening process, and how the dynamic regulation of light-associated pigments is involved in fruit ripening. Much effort should be directed to finding new molecular probes for monitoring hormone biosynthesis and responses and pigment profiles in real time in different tissues and processes.

Low temperatures interact with light signals to regulate anthocyanin accumulation through the PIF/HY5 switch in *Arabidopsis* [59, 72]. In tomato, low temperatures induce carotenoid biosynthesis [73]. Light and HY5 constitute a fruit color-change regulatory module, and regulation of tomato fruit colorization by low temperature may therefore partially rely on the light–HY5 signaling pathway. Low temperatures promote anthocyanin accumulation in summer fruits like grape and autumn fruits like apple and pear, resulting in nighttime low temperatures activating anthocyanin biosynthetic genes and MBW complex transcription, with less influence of ethylene or ABA [73–77]. Low temperatures also regulate the accumulation of anthocyanin through the cold-hardiness master regulator C-repeat binding factor (CBF), which interacts with the MBW complex to enhance its transcriptional activity in a signaling pathway probably independent of ethylene [78]. CBF promotes apple fruit softening by directly transactivating the expression of *POLYGALACTURONASE1* (*PG1*), which encodes a cell wall remodeling enzyme that plays vital roles in fruit softening [79]. Low temperatures promote sugar accumulation and softening in kiwifruit [80, 81] by inducing the expression of genes associated with cell wall remodeling and starch degradation, and also regulate degreening in lemon (*Citrus limon*) peel by upregulating chlorophyll degradation genes [82]; all these processes are independent of endogenous ethylene. More detailed experiments are needed to determine whether these processes are regulated by cold-associated CBF proteins.

### Transcriptional regulation of fruit ripening involving both ethylene-dependent and -independent processes

RIN targets a large number of ripening-related genes [24, 83], hundreds of which are up- or downregulated in the *rin* mutant. Promoter analysis shows that some of these genes possess the RIN target C-A/T-rich-G (CArG) box along with the ethylene response element (ERE), and ethylene induces expression of these genes. This reveals that RIN controls fruit ripening in an ethylene-dependent way through regulating expression of these genes. Expression of genes containing only CArG boxes cannot be induced by ethylene, implying that RIN regulates fruit ripening by directly targeting these genes independently of ethylene [24, 83]. FUL1/2 and TAGL1 interact with RIN and regulate fruit ripening in an ethylene-dependent and -independent way [28, 29]. In tomato, seed maturation and the liquefaction of locular tissue surrounding the seeds occur prior to fruit pericarp ripening and the ethylene burst; *RIN*, *NOR*, and other ripening-related genes are first induced in locular tissue and later in the pericarp [5]. Indeed, high-resolution spatiotemporal transcriptome analysis reveals that tomato fruit ripening starts from the internal tissue at the mature green stage, radiating outward. Concurrently, fruit ripening also proceeds along a latitudinal gradient starting from the bottom (style end) and proceeding to the top (stem end) [84]. High-resolution spatiotemporal transcriptome analysis identified several new TFs downstream of the master regulators RIN and NOR. *SlGRAS38*, encoding a GRAS family TF, is co-expressed in the same module (M6) as *RIN* and other well-studied ripening-related genes. Most of the genes in the M6 module were previously identified as direct/indirect targets of RIN. Silencing of *SlGRAS38* reduced carotenoid and ethylene production in ripening fruits and caused downregulation of many M6 module genes, suggesting that SlGRAS38 acts as a central regulator downstream of RIN to promote fruit carotenoid and ethylene metabolism [84]. The tomato softening-specific TF LATERAL ORGAN BOUNDRIES (SlLOB1), which specifically regulates cell wall remodeling-associated genes, was identified from transcriptome data of liquefied locular tissue and pericarp at the mature green stage [85]. Fruits with repressed *SlLOB1* expression exhibit small amounts of liquefied locular gel, delayed softening, and severely downregulated expression of the cell wall-loosening gene *EXPANSIN1* (*EXP1*), a direct target of SlLOB1 [85]. *SlLOB1* RNAi lines display no difference from control fruits in ethylene production from ripening initiation until the medium ripening stage but accumulate more ethylene in the late ripening stage, indicating that *SlLOB1* RNAi fruits might accumulate more carotenoids through ethylene regulation in the late ripening stage. In the early ripening stage, RIN and NOR may transactivate *SlLOB1* in locular tissue to regulate fruit pericarp softening in an ethylene-independent manner [85].

Intriguingly, re-analysis the central hub genes of the M6 module identified two bZIP family TFs (Solyc01g104650 and Solyc02g092620) and nine unknown proteins (Solyc02g092630, Solyc03g095880, Solyc03g116630, Solyc04g016460, Solyc06g064900, Solyc09g011470, Solyc10g024430, Solyc10g080670, and Solyc12g007190) [84]. Given that RIN forms a central regulator for co-expressed genes in the M6 module, these TFs and unknown proteins may be regulated by, and/or direct targets of, RIN, and act as potential novel regulators in fruit ripening.

These high-resolution spatiotemporal transcriptome data were integrated into the Tomato Expression Atlas (TEA; http://tea.solgenomics.net/) database, allowing any of the gene expression profiles to be visualized at different stages and in different tissues [84]. In tomato, several quantitative trait loci (QTLs) have been proved to control the synthesis of flavor-related chemicals, including sugars, acids, and volatiles [86–90]. These QTLs were validated as encoding key enzymes for flavor-related components metabolism; however, knowledge of the transcriptional regulatory mechanism remains limited. Most of the QTLs are co-expressed in the M6 module, indicating that the TFs in the M6 module may participate in regulating flavor biosynthesis. Considering that flavor is one of the key agronomical traits that determine fruit taste and commercial value, these TFs should be investigated further to understand the genetic regulation of flavor biosynthesis.

### Epigenetic regulation and fruit ripening

**Figure 2:**
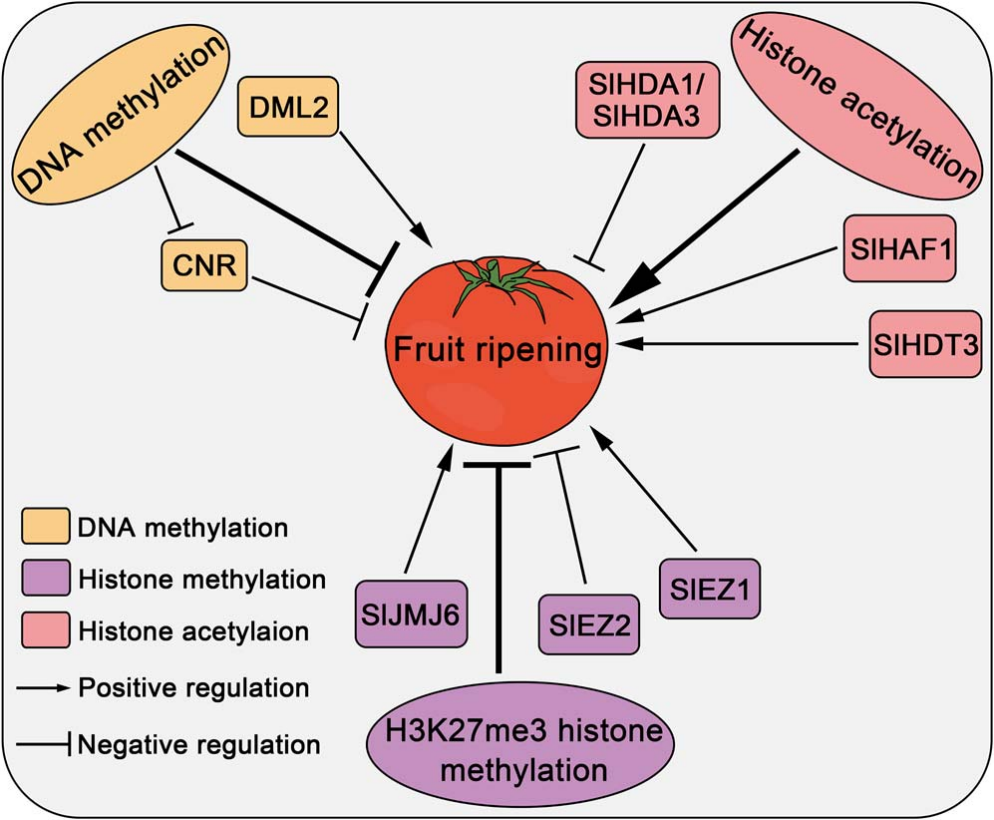
Roles of epigenetic modifications in tomato fruit ripening. DNA methylation, histone methylation, and acetylation are the main epigenetic modifications influencing fruit ripening. DNA methylation and H3K27me3 histone methylation are repressive epigenetic marks. DML2, a DNA demethylase, induces DNA demethylation to promote fruit ripening. Hypermethylation of the promoter region of *CNR* represses its expression and further inhibits fruit ripening. The histone methyltransferases EZ1 and EZ2 play distinct roles during tomato ripening. SlJMJ6, a histone demethylase, facilitates tomato fruit ripening. HDT3 and HDA1/HDA3 are histone deacetylases playing contrasting roles in tomato fruit ripening. HAF1, a histone acetyltransferase, may facilitate tomato fruit ripening.

Recent studies have revealed that fruit ripening is not regulated only by TFs and hormones; epigenetic modifications also play important roles in the fruit ripening process (Fig. 2). An increasing amount of evidence indicates that heritable changes in gene expression can be caused by environmental factors through epigenetic mechanisms [91–93]. In eukaryotes, chromatin carries the genetic and regulatory information of an organism, and the structure of chromatin and the information it carries directly affect the regulation of gene transcription. By regulating chromatin structure through methylation or histone post-translational modifications (PTMs), epigenetic processes can directly affect gene expression in subsequent generations. Epigenetic regulation of genes can occur through multiple pathways, some of which play important roles in fruit ripening [5, 93, 95].

### DNA methylation

DNA methylation refers to the addition of a methyl group, provided by *S*-adenosine methionine, to the carbon 5 of cytosine in a reaction catalyzed by methyltransferase [93]. DNA methylation is maintained by several methyltransferases, such as DNA METHYLTRANSFERASE 1 (MET1), DECREASED DNA METHYLATION 1 (DDM1), CHROMOMETHYLASE 3 (CMT3), CMT2, and others [96]. Meanwhile, methylation of plant DNA can be reversed by a series of demethylases, such as DEMETER (DME), DEMETER-LIKE 2 (DML2), DML3, and REPRESSOR OF SILENCING 1 (ROS1) [97]. The activities of methyltransferases and demethylases determine DNA methylation levels [98, 99].

Many studies have demonstrated that DNA methylation of ripening-associated genes can affect fruit ripening. As mentioned earlier, the *cnr* phenotype results from hypermethylation of a 286-bp contiguous region located 2.4 kb upstream of the first ATG of the *CNR* gene [16]. In addition, *VITAMIN E 3* (*VTE3*) expression as well as the vitamin E content of ripe tomato fruits are affected by DNA methylation of a SINE retrotransposon located in the promoter region of the *VTE3* gene encoding 2-methyl-6-phytylquinol methyltransferase in the vitamin E biosynthetic pathway [100]. Evidence from other fruit species also demonstrates that fruit ripening is regularly associated with DNA methylation. For example, differential methylation of the *MYB10* promoter induces different levels of *MYB10* transcription, resulting in differences in anthocyanin accumulation and variable color patterns in the peels of apple and pear [92, 101–103]. A recent study also demonstrated that decreased DNA methylation increases the transcription level of peach *TERPENE SYNTHASE 3* (*PpTPS3*) and flavor-related linalool accumulation during peach fruit ripening [104]. In short, DNA methylation plays an important role in fruit ripening, but its regulatory mechanisms need further study.

In addition to DNA methylation, DNA demethylation also affects fruit ripening. For example, the DNA methyltransferase inhibitor 5-azacytidine accelerates tomato fruit ripening [25]. The promoters of numerous ripening-related genes are demethylated during fruit ripening, and RIN frequently binds to these demethylated regions [25]. There are two pathways for DNA demethylation: active DNA demethylation and passive DNA demethylation. Active DNA demethylation is initiated by demethylases, while passive DNA demethylation refers to a decrease in DNA methyltransferase concentration or activity, resulting in loss of DNA methylation in newly biosynthesized strands [105]. In tomato, fruit ripening is accompanied by a global decrease in DNA methylation caused by upregulation of *SlDML2*, which encodes a tomato demethylase; thus, knockout of *SlDML2* results in green ripe fruits [94, 106]. Moreover, SlDML2 is involved in the expression of many genes associated with fruit ripening, including *RIN*, *SlACS4*, and *SlPSY1*, as well as cell wall remodeling genes [94]. Passive DNA demethylation also plays an important role in the process of fruit ripening. Knockdown of the DNA methyltransferase *SlMET1* in fruit of the *cnr* mutant partially restores tomato non-ripening phenotypes in ~30% of fruits, indicating that SlMET1 leads to hypermethylation of ripening-related genes in the *cnr* mutant, thus inhibiting fruit ripening [107].

DNA methylation has also been studied in non-climacteric fruits. Strawberry (*Fragaria vesca*) undergoes DNA hypomethylation during fruit ripening, but rather than being caused by upregulated expression of DNA demethylase genes, this is caused by downregulation of RNA-directed DNA methylation (RdDM) pathway genes during strawberry fruit ripening [108]. Moreover, treating unripe strawberry fruits with a DNA methylation inhibitor causes early fruit ripening [108]. This evidence suggests that DNA hypomethylation is of great importance to fruit ripening; however, not all fruit ripening is accompanied by DNA hypomethylation. Sweet orange (*Citrus sinensis*) exhibits a global increase in DNA methylation during fruit ripening, which is likely caused by decreased DNA demethylase activity [109]. Moreover, treating unripe orange fruits with a DNA methylation inhibitor delays ripening, suggesting that DNA hypermethylation is of great importance to orange fruit ripening [109]. In conclusion, DNA methylation and demethylation are both important for fruit ripening, but their roles in ripening vary among fruit species.

### Histone post-translational modification

The amino terminus of histones can be modified by methylation, acetylation, phosphorylation, ubiquitination, etc. [110]. These histone modifications impact chromatin conformation and thus regulate gene expression. Furthermore, increasing evidence indicates that histone modifications play critical roles during fruit ripening.

The histone methylation state is mediated by histone methyltransferases (HMTs) and histone demethylases (HDMs). Histone methylation mainly occurs on lysine and arginine residues of histones H3 and H4 [8]. This histone methylation is associated with gene expression and involved in fruit ripening. In plant genomes, dimethylation at lysine 9 of histone H3 (H3K9me2) marks regions of heterochromatin where transposons and repeats are enriched, and trimethylation of histone H3 at lysine 27 (H3K27me3) mediated by the polycomb repressive complex 2 complexes (PRC2s) is associated with gene repression [92, 111, 112]. There is evidence that H3K27me3 has a negative effect on the initiation of ripening [32]. ENHANCER OF ZESTE, referred to as E(z), is an HMT and part of the core element of PRC2s. Two genes encoding E(z) protein in tomato, *SlEZ1* and *SlEZ2*, have different functions in fruit development and ripening [113, 114]. Knockdown of the *SlEZ1* gene causes an increase in fruit locule number and altered flower morphology but has no effect on fruit size or color [113]. By contrast, knockdown of the *SlEZ2* gene leads to modifications in carpel initiation and fruit cuticle formation during fruit development and ripening [113]. In addition to HMTs, HDMs also play important roles in fruit ripening. SlJMJ6 activates the expression of ripening-related genes by removing their H3K27 methylation, thus facilitating tomato fruit ripening [115]. In peach and apple, NACs regulate ester formation by activating *ALCOHOL ACYLTRANSFERASES 1* (*AAT1*) gene expression during fruit ripening. Additionally, the removal of histone mark H3K27me3 from *NAC* and *AAT* genes also increases their expression [116].

Acetylation and deacetylation of histones are catalyzed by histone acetyltransferases (HATs) and histone deacetylases (HDACs) [117]. Several studies have shown that both of these enzymes are involved in fruit ripening. For instance, expression of the *SlHAF1* gene encoding a tomato HAT peaks in tomato fruit 10 days after breaking, implying an important role in the process of tomato fruit ripening [118]. Similarly, expression of two orange genes, *CsHAF1* and *2*, also peaks at the mature stage of orange fruits (240 days after flowering), suggesting important roles in orange fruit ripening [119]. Repression of the tomato HDAC gene *SlHDT3* suppresses ethylene biosynthesis and carotenoid accumulation, thus delaying tomato fruit ripening [120]. Conversely, repression of the other two HDAC genes, *SlHDA1* and *SlHDA3*, increases carotenoid accumulation and ethylene content, thus promoting tomato fruit ripening [121, 122]. The orange HDAC gene *CsHDA5* displays increasing expression levels during development of orange fruit, implying that CsHDA5 may also participate in orange fruit development by repressing gene expression [119]. Similarly, the banana HDAC protein MaHDA1 suppresses an ethylene biosynthetic gene by interacting with an ethylene negative regulator, ERF11, during fruit ripening [40]. A recent study by Vall-Llaura *et al*. [123] explored the role of an HDAC gene, denoted *SIRTUINS 2* (*SRT2*), in pear ripening, finding that PbSRT2 appears to regulate pear fruit ripening by mediating sugar metabolism. In summary, many HATs and HDACs from a variety of fruits are involved in fruit ripening, but the precise mechanisms by which they regulate fruit ripening remain unknown and require further study.

### N^6^-Methyladenosine mRNA modification in fruit ripening

**Figure 3:**
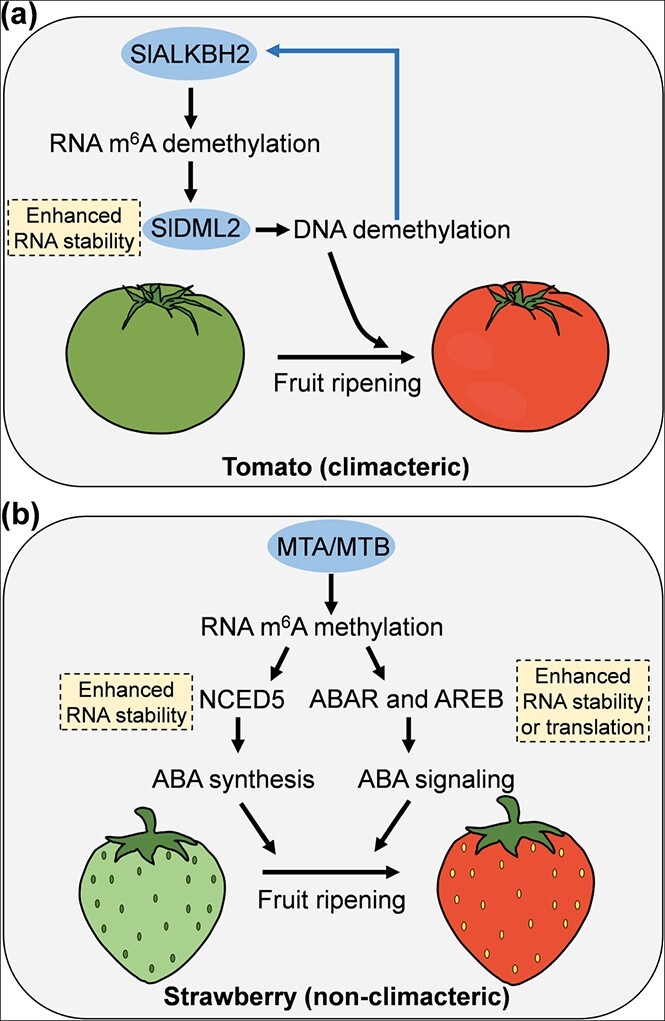
Proposed model for m^6^A-induced ripening in tomato and strawberry fruits. **a** In climacteric tomato, SlALKBH2, an m^6^A demethylase, demethylates the m^6^A modifications on *SlDML2* mRNA, which encodes a DNA demethylase gene required for tomato fruit ripening. SlDML2 can in turn regulate SLALKBH2 expression though DNA 5mC demethylation. **b** In non-climacteric strawberry fruit, m^6^A methyltransferases MTA and MTB target NCED5, ABAR, and AREB1 in the ABA biosynthesis or signaling pathway, enhancing mRNA stability of *NCED5* and *AREB1* as well as translation of *ABAR*; therefore, enhanced ABA biosynthesis and signaling positively regulate the ripening of strawberry fruit.

N^6^-Methyladenosine (m^6^A) is the most prevalent mRNA modification in eukaryotes and, in turn, moderates almost all aspects of RNA metabolism, including mRNA stability, translation efficiency, splicing, nuclear retention, nuclear export, and 3′-end processing [124, 125]. Recent studies have demonstrated that m^6^A plays profound roles in fruit ripening (Fig. 3). In tomato, mRNA m^6^A methylation displays similar dynamic changes to DNA 5-methylcytosine (5mC) methylation in the progression of fruit ripening, suggesting a correlation between these two nucleic acid modifications. Mutation of *SlALKBH2*, encoding an m^6^A RNA demethylase, produces a delayed ripening phenotype with obviously increased proportions of m^6^A modification, indicating that SlALKBH2 is required for fruit ripening in tomato. Indeed, SlALKBH2 directly demethylates the m^6^A of the DNA demethylase gene *SlDML2* to increase its stability, and SlDML2 in turn regulates *SLALKBH2* expression though DNA 5mC demethylation [126]. These findings reveal a feedback loop between m^6^A mRNA modification and DNA methylation that regulates tomato fruit ripening (Fig. 3a).

Transcriptome-wide mapping of m^6^A uncovered RRACH as the most prevalent motif in the fruit ripening process of strawberry, a typical non-climacteric fruit, distinct from the UGUA motif found in tomato ripening [126, 127]. However, the mechanisms underlying this target specificity of m^6^A in different fruits remain to be further explored. At ripening onset of strawberry, the proportion of m^6^A is considerably increased in the coding sequence (CDS) region, especially adjacent to the start codon, accompanied by a decreased percentage of m^6^A enrichment in the 3′ region, showing a dramatic change in m^6^A pattern from that before ripening. This is different from tomato, which shows little change in m^6^A distribution pattern with the onset of ripening, with ~88% m^6^A modification highly enriched around the 3′ region [126, 127]. This also raises an intriguing question of how the m^6^A distribution pattern is shaped at ripening onset in strawberry and reflects the divergent roles of m^6^A during strawberry and tomato ripening. m^6^A modifications mediated by m^6^A methyltransferases enhance mRNA stability of the ABA biosynthesis gene *9-cis-epoxycarotenoid dioxygenase 5* (*NCED5*) and *AREB1* as well as translation of *putative ABA receptor* (*ABAR*) in the ABA biosynthesis or signaling pathways, positively regulating the ripening of strawberry fruit (Fig. 3b). Together, these findings demonstrate a functional link between the m^6^A-mediated ABA pathway and strawberry fruit ripening [127]. Given that the m^6^A genome-wide consensus motifs, distribution pattern, and the association with gene expression are evolutionarily divergent in ripening of climacteric and non-climacteric fruits, species-specific regulators may exist. Future studies should therefore pay more attention to identify these regulators.

## Conclusions and perspectives

Fruit ripening is a complex, genetically programmed, and environmentally regulated process and is coordinated through the combined effects of plant hormones, TFs, and epigenetic modifications. Taking tomato as example, fruit ripening starts from the mature green stage in the internal tissue. Expression of *RIN* and other TF genes is upregulated through DNA demethylation and removal of histone H3K27me3 methylation. RIN together with other TFs then induces ethylene biosynthesis to regulate almost every aspect of ripening. These TFs are also stimulated by ethylene signals, creating a positive feedback regulatory circuit. Epigenetic modifications, ripening-related TFs, and hormones thus constitute a regulatory circuit that dynamically regulates the tomato fruit ripening process. This working model may serve as a guide when studying the mechanisms regulating ripening in other fruit species.

High-resolution spatiotemporal transcriptome analysis revealed that gradients of gene expression in different tissues and at different stages may modulate fruit ripening-related traits. Sugar, acid, and aroma production are key agronomical traits in fruit crops. Therefore, new TFs associated with those phenotypes should be further investigated.

The initiating ripening signals remain unknown. The cell–cell communication in developmental tissues is generally mediated by plasma membrane-resident receptor-like kinases (RLKs) or receptor-like proteins (RLPs). Upon perceiving the extracellular signal molecules, these receptors transduce the signals to the cytoplasm to regulate cellular activities [128]. RLKs and RLPs have been extensively studied in immunity, sexual reproduction, and seed development but rarely reported in fruit development and ripening. Several studies suggest that RLKs and peptide hormones regulate fruit ripening [129–131], but the mechanisms remain unknown. There is a possibility that the peptides from mature seeds trigger fruit ripening initiation via RLKs and downstream signaling transduction.

## Acknowledgements

The authors are grateful to the authors of the excellent papers discussed. The authors also apologize to the authors of the other excellent papers in this area that could not be discussed because of lack of space. This work was supported by National Key Research and Development Program of China (No. 2020YFA0907600), the National Natural Science Foundation of China (No. 31730102 to A.Z. and 32000184), the Natural Science Foundation of Shandong Province (No. ZR2020QC023), and the China Postdoctoral Science Foundation (No. 2020 M672093).

## Author contributions

X.L., A.Z., and C.-X.Y. planned and designed this review paper. X.L., X.W., and A.Z. prepared and drafted the manuscript. X.L. and C.-X.Y. revised the manuscript. Y.Z. checked the grammar and helped to improve the English.

## Conflict of interest

The authors declare no conflicts of interest.
